# Penetration ability and microhardness of infiltrant resin and two pit and fissure sealants in primary teeth with early enamel lesions

**DOI:** 10.1038/s41598-022-08725-9

**Published:** 2022-03-17

**Authors:** Mahtab Memarpour, Arefe Abedinzade, Azade Rafiee, Atieh Hashemian

**Affiliations:** 1 Oral and Dental Disease Research Center, Department of Pediatric Dentistry, School of Dentistry, Shiraz University of Medical Sciences, Shiraz, Iran; 2 Student Research Committee, Department of Pediatric Dentistry, School of Dentistry, Shiraz University of Medical Sciences, Shiraz, Iran; 3 Department of Dental Biomaterials, School of Dentistry, Tehran University of Medical Sciences (TUMS), Tehran, Iran

**Keywords:** Health care, Medical research, Materials science

## Abstract

To determine the penetration depth and enamel microhardness (EMH) of an infiltrant resin and two fissure sealants in primary teeth with early enamel lesions. We randomly divided 174 sound teeth into six groups (n = 29): (1) phosphoric acid (PA) + Clinpro, (2) PA + Aegis, (3) Icon, (4) hydrochloric acid (HCl) + Clinpro, (5) HCl + Aegis, and (6) control. Percentage penetration (%PP) was analyzed by confocal laser scanning microscopy (n = 15). EMH was measured (n = 12), and the percentage of EMH recovery (%REMH) was calculated. Twelve samples were examined under a scanning electron microscope (SEM). All data were analyzed with the Kruskal–Wallis and one-way ANOVA tests (p < 0.05). Groups 3 and 4 showed the highest %PP (all, p < 0.05). Icon application led to significantly higher %REMH compared to the others (p < 0.05). Groups 2 and 5 showed the lowest reduction in %REMH after pH-cycling. Application of Icon and Clinpro with HCl pretreatment showed the greatest %PP. pH-cycling led to a decrease in %REMH for all of the materials, although this effect was lower in teeth treated with Aegis.

## Introduction

Early childhood caries (ECC) begin on smooth buccal surfaces in primary anterior teeth. Although the lesion is initially non-cavitated, it can progress to a cavity. Micro-invasive methods are proposed to stop progression to a cavity. Overall, these techniques include sealing the lesion and resin penetration into enamel, which is porous because of demineralization^1, 2^.

Fissure sealant (FS) is a common method to arrest incipient caries by pretreatment with phosphoric acid and application of a resin-based sealant material^2^. The use of infiltrant resin (IR) is another approach where a low viscosity resin penetrates by capillary force into the subsurface lesion and occludes the porous area. Both approaches can halt caries progression^3–5^. The results of some studies have revealed that the superficial surface of the enamel reduced resin impregnation because of a decrease in “pore volume”. Pretreatment with a 15% hydrochloric acid gel for 2 min compared to 37% phosphoric acid more effectively removed the highly mineralized superficial barrier and eroded the enamel surface, which improved resin penetration into the subsurface area^4, 6^.

Some studies on permanent teeth showed the advantages of pretreatment with 15% hydrochloric acid rather than 37% phosphoric acid to increase resin-based material penetration into non-cavitated early caries lesions^4, 7, 8^. Also IR can penetrate to the body of the lesion and increase enamel microhardness (EMH) more than conventional sealant materials^8^. However, it is still unclear whether IR is effective in primary teeth. Stereomicroscope observation showed increased penetration of IR in primary teeth compared to permanent teeth^9^. In addition, Swamy et al. assessed the extent of IR penetration into white spot lesions of primary teeth enamel. They concluded that IR is a predictable material, which can deeply seal porosities in white spot lesions of primary teeth enamel^10^. IR can mask the appearance of white spot lesions from light reflection, which is similar to sound enamel^9, 11^.

We designed this research by taking into consideration the importance and benefits of minimal intervention dentistry, especially in children, and the availability of FS compared to IR in pediatric dentistry. We hypothesized that FS combined with phosphoric acid or hydrochloric acid would be similar or superior for deeper penetration or increasing microhardness compared to IR for early caries lesions because of the pretreatment methods and the effect on enamel. The null hypothesis was tested against an alternative hypothesis that differences in penetration or microhardness would be found between the materials. To date, no studies have assessed these microinvasive methods and compared them in primary teeth. The aim of this study was to evaluate microinvasive techniques (i.e., sealing and infiltration) as treatment for early caries lesions in primary teeth. We compared the efficacy of IR and two resin-based FS that contained fluoride or amorphous calcium phosphate (ACP) to penetrate into subsurface lesions and alter microhardness. Confocal laser scanning microscopy (CLSM), EMH, and scanning electron microscope (SEM) evaluations were compared after the intervention.

## Materials and methods

This research protocol was conducted in accordance with the principles stated by the Human Ethics Review Committee of the School of Dentistry, Shiraz University of Medical Sciences. In this study, we used extracted, sound primary anterior teeth (n = 185). All parents provided their written informed consent for the use of their children’s teeth. The roots of the teeth were removed and the specimens were cleaned by a rotating brush and disinfected by immersion in 0.1% chloramine T solution for one month. The samples were kept in distilled water at 37 °C. Prior to beginning the experiment, the enamel of each tooth was observed under a stereomicroscope in order to exclude teeth with any defective enamel, microcracks or staining. The EMH values for the incisal, middle, and cervical thirds of the teeth were measured to ensure acceptable baseline microhardness. Totally, 11 teeth did not fulfill the inclusion criteria. Then, we randomly divided 174 teeth into six groups (n = 29). The teeth were allocated to three treatment groups (IR, resin-based FS containing fluoride, and ACP with different pretreatments [37% phosphoric acid or 15% hydrochloric acid]). Then, the teeth were mounted in polyester (Pooyesh Sanat, Qazvin, Iran). The labial surface was placed on a parallel surface with the mold. Each tooth surface was polished with 600-, 800-, and 2400-grit waterproof silicon carbide paper and 0.5–3 μm aluminum oxide in order to generate a flat, glossy and smooth surface. Next, the teeth were washed for 20 s in distilled water. Then, the specimens in each group were randomly divided into three sub-groups to assess CLSM (n = 15), EMH (n = 12) and SEM (n = 2). The samples also underwent demineralization.

### Sample preparation before enamel microhardness (EMH)

We measured EMH in four steps: baseline (before demineralization), after demineralization, after intervention, and seven days after pH-cycling. A 3 × 4.5 mm window was created on the labial surface of the tooth with a paper label. Two layers of nail polish were applied to cover the rest of the tooth’s surface. The exposed area was divided into three similar size sections, where 1/3 of the incisal side was used to measure EMH at baseline, 1/3 of the middle to assess EMH after intervention and pH-cycling, and 1/3 of the cervical area was used to measure EMH after demineralization. In order to avoid any interference between each step and to accurately calculate EMH of these parts, we covered the area with nail polish before each step. First, the 1/3 incisal area was covered with a different color nail polish and then the samples were demineralized.

### Early caries lesions

Each tooth was soaked in 30 mL demineralization solution at 37 °C for 96 h. The solution contained 0.1 mM lactic acid solution, 3 mM CaCl_2_, 3 mM KH_2_PO_4_, and 0.2% guar gum. The final pH was adjusted to 4.5 with 50% sodium hydroxide. The solution was replaced by fresh solution after 48 h. After 96 h, we washed each sample with deionized water for 20 s and allowed them to air dry^12^.

Next, the 1/3 cervical area of each EMH specimen was covered by nail polish and the following interventions were performed on all the teeth.

### Experimental groups

*Group 1 (phosphoric acid + fluoride containing FS)* The enamel was etched with a 37% phosphoric acid gel (3M ESPE, St. Paul, MN, USA) for 30 s, rinsed for 30 s, and dried for 30 s. Sealant (Clinpro™, 3M ESPE, St. Paul, MN, USA) was applied according to the manufacturer’s instructions on the treated surface and an explorer was used to prevent void formation. Next, a halogen light cure unit (Coltolux, Coltene, Whaledent, Altstätten, Switzerland) at a power density of 550 mW/cm^2^ was used for 40 s to polymerize the FS at a 1 mm distance to the surface.

*Group 2 (phosphoric acid + ACP-containing FS)* The tooth surface was etched, washed, and dried as previously mentioned. Then, ACP-containing FS (Aegis®, Bosworth, Keystone Industries, Gibbstown, NJ, USA) was applied onto the treated surface according to the manufacturer’s instructions and then light-cured for 40 s.

*Group 3 (hydrochloric acid + IR)* The enamel was etched with a 15% hydrochloric acid gel (Icon etch®, DMG, Hamburg, Germany) for 2 min, washed for 30 s, and dried. The samples were dehydrated with 99% ethanol (Icon Dry®, DMG, Hamburg, Germany) for 30 s and gently dried. Next, IR (Icon®, DMG, Hamburg, Germany) was applied over the treated surface for 3 min, and the excess was removed, then light-cured for 60 s. Icon was reapplied for 1 min and light-cured for 60 s. All the procedures were performed according to the manufacturer’s instructions.

*Group 4 (hydrochloric acid + fluoride containing FS)* The enamel surface was etched with 15% hydrochloric acid for 2 min, then the surface was washed for 30 s and dried. A Clinpro™ sealant was applied over the surface for 3 min and the surface was light-cured for 60 s.

*Group 5 (hydrochloric acid + ACP-containing FS)* The procedure performed was similar to group 4; however, Aegis was used as the sealant material.

*Group 6 (control)* No intervention was used and the teeth only underwent pH-cycling.

### Confocal laser scanning microscopy (CLSM) assessment

The samples were prepared prior the CLSM assessment. First, the enamel surface was etched with phosphoric acid or hydrochloric acid as previously described. Then, the tooth was washed in deionized water for 30 s and dried for 30 s. Each specimen was immersed in 0.1% ethanolic tetramethyl-rhodamine isothiocyanate (Merck, Darmstadt, Germany) for 12 h and dried for 10 s. Next, the materials were applied onto the treated surface as previously described. In order to obtain a 1 mm section, each sample was sectioned in the buccolingual direction across the center of the lesion with a diamond saw (Mecatome, Presi, Eybens, France) and continuous water irrigation. The slice was fixed on a slide and polished to 0.5 ± 0.2 mm thickness by 1000-, 2400-, 3000-, and 4000-grit waterproof silicon carbide paper. The samples were then immersed in 30% hydrogen peroxide for 12 h to remove any unbound red fluorophore, and then washed for 10 s. Next, the section was dried for 20 s and subsequently placed in a 50% ethanol solution of 100 μM fluorescein sodium (Merck, Darmstadt, Germany) for 3 min in order to identify any porous parts that were not filled by the materials. Each sample was washed with deionized water for 10 s and dried^13, 14^.

### Image analysis

**Figure 1:**
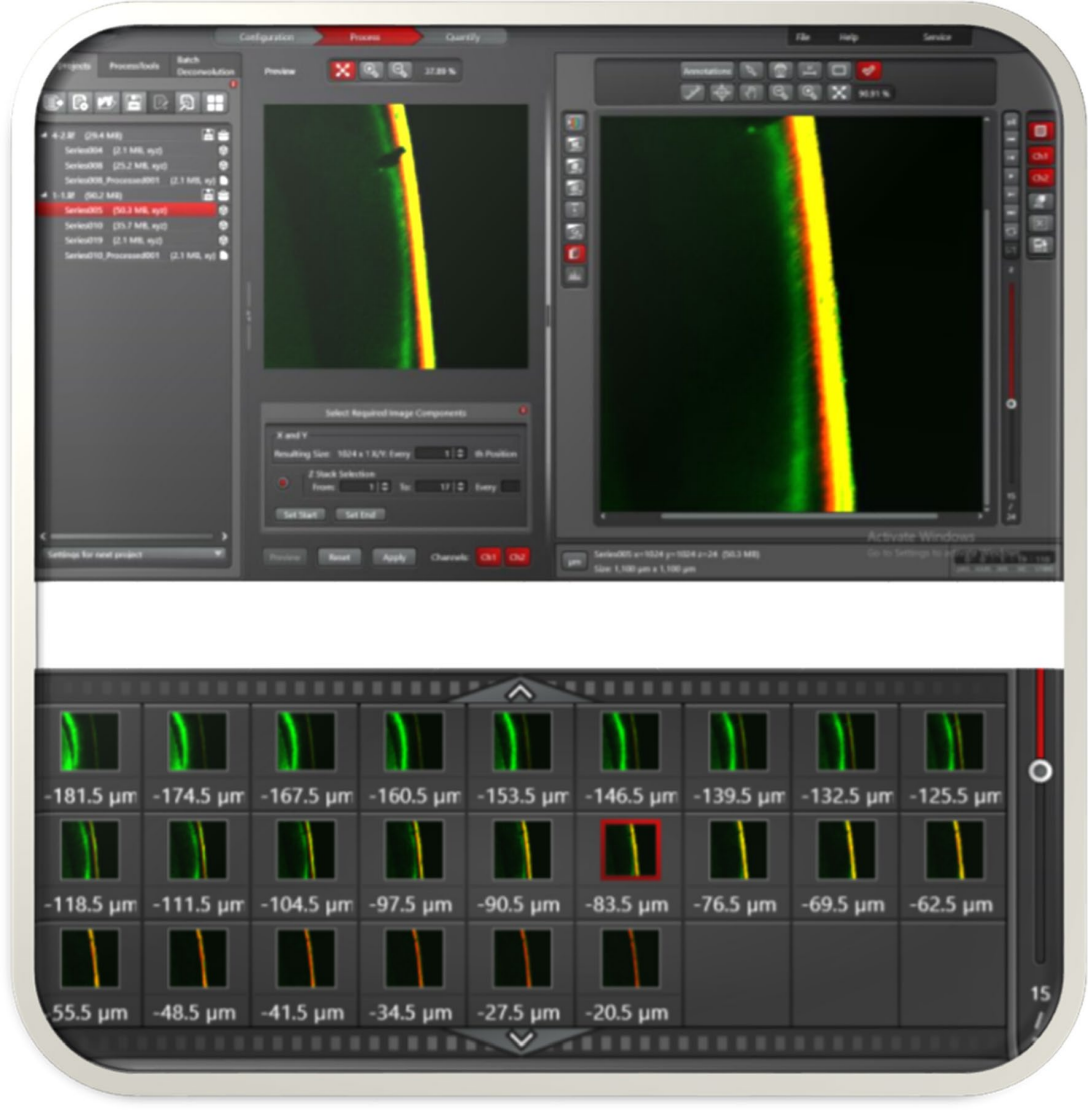
Examination of Z-stack images (3D) to select the most suitable single-plane according to LAS X 3D Visualization (Leica Microsystems GmbH, Wetzlar, Germany).

A 10 × objective in a dual-fluorescence mode was used to assess the samples. Z-stack images (3D) at 100–150 µm thicknesses were examined in order to select the most suitable single-plane image that was a 1024 × 1024-pixel with 1100 × 1100 µm field of view according to LAS X 3D Visualization (Leica Microsystems GmbH, Wetzlar, Germany). ImageJ software (National Institute of Health, Bethesda, MD, USA) was used for quantitative analyses of the selected 2D CLSM images (Fig. 1). For each image, the lesion depth (LD) and the sealant/infiltrant penetration depth (PD) were measured from the enamel surface at nine selected points that were located 20 µm apart and the average was recorded. The vertical distance from the enamel surface to the deepest front of the porous enamel (green area) was defined as the LD. The maximum vertical distance from the enamel surface to the deepest penetrated area (red color) was considered to be the PD. Percentage of penetration (%PP) of materials into the lesion was calculated as PP = PD/LD × 100.

### Microhardness test

After intervention, we removed the nail polish from the 1/3 incisal and cervical areas, and these samples were sectioned in the buccolingual direction across the center of the lesion to obtain two halves of each tooth. Cross-sectional EMH was measured at the interface of the material enamel and 50 µm above and below this area. The deep EMH in all teeth were measured with a Vickers diamond indenter (MHV-1000Z, SCTMC, China) at a 50 g load for 10 s at three points per sample. Teeth that had a baseline EMH between 223.49 and 243.03 Vickers hardness number (VHN) were selected for this study. Then, the cross-sections of the samples were covered with nail polish and pH-cycling was performed. The demineralization-remineralization solutions only contacted the buccal surface of the teeth. After the acidic challenge, we removed the nail polish and deep EMH was measured at the 1/3 middle of each tooth. The percentage of EMH recovery (%REMH) in the remineralized enamel was determined as follows:

$$\% {\text{REMH}}_{{{\text{Intervention}}}} = \, \left( {{\text{VHN}}_{{{\text{Intervention}}}} {-}{\text{ VHN}}_{{{\text{Demineralization}}}} } \right) \, / \, \left( {{\text{VHN}}_{{{\text{Baseline}}}} {-}{\text{ VHN}}_{{{\text{Demineralization}}}} } \right) \, \times { 1}00$$

$$\% {\text{REMH}}_{{\text{pH-cycling}}} = \, \left( {{\text{VHN}}_{{\text{pH-cycling}}} {-}{\text{ VHN}}_{{{\text{Demineralization}}}} } \right) \, / \, \left( {{\text{VHN}}_{{{\text{Baseline}}}} {-}{\text{ VHN}}_{{{\text{Demineralization}}}} } \right) \, \times { 1}00$$

### pH-cycling

The following pH-cycling protocol was implemented to simulate daily pH changes in the oral cavity. The tooth samples were individually subjected to a daily series of six, one-hour immersion periods in demineralization solution and six, two-hour intervals in remineralizing solution. For the remainder of the day, the samples were left in the remineralizing solution. This cycle was repeated daily for 28 days. Both solutions were refreshed at the beginning of each daily cycle^15^.

The demineralization solution contained 0.1 mM lactic acid solution, 3 mM CaCl_2_, 3 mM KH_2_PO_4_, and 0.2% guar gum. The final pH was adjusted to 4.5 with 50% sodium hydroxide.

### Remineralizing solution

The solution consisted of 2.200 g/L gastric mucin, 0.381 g/L sodium chloride (NaCl), 0.213 g/L calcium chloride (CaCl_2_·2H_2_O), 0.738 g/L potassium hydrogen phosphate (K_2_HPO_4_·3H_2_O), and 1.114 g/L potassium chloride (KCl). The final pH was adjusted to 7.00 at 37 °C with 85% lactic acid. The solution was changed every 48 h.

### Scanning electron microscope (SEM) observation

We examined 12 samples (n = 2 per group) with SEM. The teeth were prepared for SEM assessment in order to observe penetration by the material and the enamel-material interface. The samples were sectioned in the buccolingual direction across the center of these materials and prepared for SEM. All SEM data were obtained with a SEM (VEGA, Tescan, Brno, Czech Republic) at 2500 × and 1000 × magnifications.

### Statistical analysis

SPSS version 22 (IBM Corp, Armonk, NY, USA) was used for data analysis. The Shapiro–Wilk test was used to assess the normality of the data distribution for CLSM. The differences between percentage of materials that penetrated were assessed by the Kruskal–Wallis H and Dunn’s post hoc tests. The depth of penetration and EMH values were analyzed with one-way ANOVA and the Tukey HSD post hoc test. The paired t-test was used to compare the %REMH after the interventions and pH-cycling. Significance was set at p < 0.05.

### Ethics approval

The study was approved by the Ethics Review Committee of the School of Dentistry, Shiraz University of Medical Sciences (IR.SUMS.DENTAL.REC.1399.152).

## Results

Totally, 7 teeth were destroyed during the preparation before CLSM assessment. The data for CLSM did not follow any normal distribution. Table 1 shows the median and interquartile range (IQR) values for LD, PD, and %PP. Group 3 had a significantly greater %PP compared to groups 1, 2 (both, p < 0.001), and 5 (p = 0.002). Although CLSM images showed additional deep penetration in group 3, there was no significant difference between groups 3 and 4 (p = 0.922). The %PP was significantly greater in group 4 compared with groups 1 (p = 0.008) and 2 (p < 0.001), but was not statistically different compared to group 5 (p = 0.468). Group 5 had a greater %PP in comparison with group 2 (p = 0.049). However, no statistically significant difference was found between groups 1 and 2 (p = 0.999). There were no significant differences found between groups 4 and 5 (p = 0.468).

**Table 1:** Comparison of LD, PD, and %PD/LD in the study groups.

Group	LD		PD		PD/LD%	
	Median	IQR	Median	IQR	Median	IQR
1 (n = 15)	174.00	34.25	78.00	11.43	44.83^AB^	4.10
2 (n = 14)	182.48	47.93	70.40	12.76	37.82^A^	8.67
3 (n = 14)	303.76	28.96	232.12	16.11	75.74^C^	8.20
4 (n = 13)	307.33	15.33	191.33	14.66	61.80^CD^	1.24
5 (n = 13)	331.10	7.00	192	9.00	58.02^BD^	1.27
6 (n = 14)	181.53	4.2	0	0	0^E^	0

*LD* Lesion depth, *PD* Sealant/infiltrant penetration depth, *%PD/LD* Percentage of PD to LD, *IQR* interquartile range. Values with the same superscript capital letter were not significantly different. Statistical significance: p < 0.05.

Group 1: Phosphoric acid + Clinpro™; group 2: Phosphoric acid + Aegis®; group 3: Icon®; group 4: Hydrochloric acid + Clinpro™; group 5: Hydrochloric acid + Aegis®; group 6: Control.

**Figure 2:**
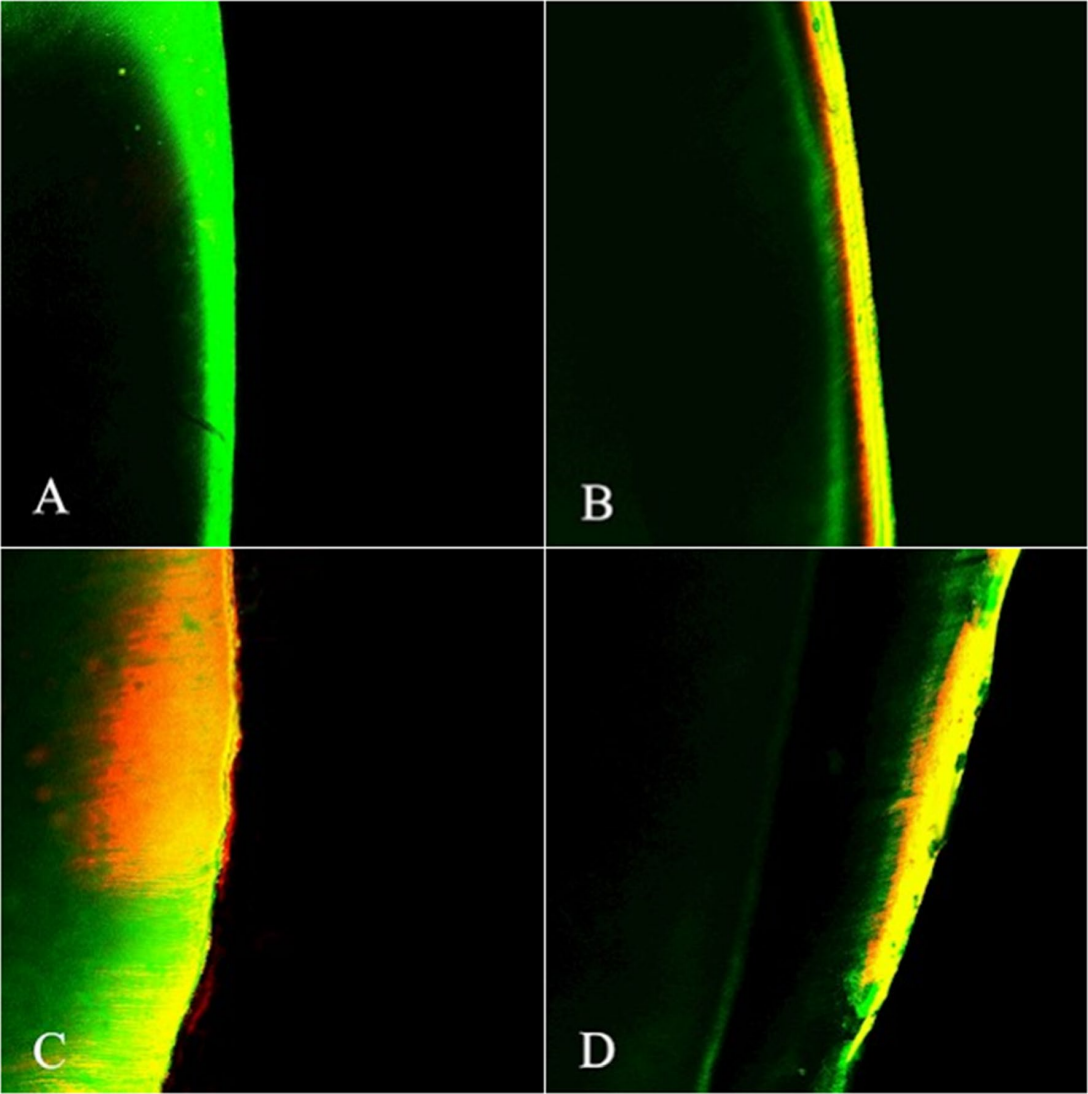
Confocal laser scanning microscopy image the enamel after: (**A**) Demineralization (control group), (**B**) Fissure sealant penetration into demineralized enamel treated with 37% phosphoric acid, (**C**) Infiltrant resin that penetrated into demineralized enamel, (**D**) Fissure sealant that penetrated into demineralized enamel treated with 15% hydrochloric acid. Red: Penetration zone of methyl-rhodamine isocyanate (diffusion zone). Green: Penetration zone of sodium fluorescein (demineralized zone).

Figure 2 shows that the sealant associated with phosphoric acid resulted in superficial penetration of the materials, which, in general, was less than those pretreated with hydrochloric acid.

Table 2 shows the mean ± SD for EMH in each group at the four time periods. At baseline, EMH ranged from 223.49 to 243.03 VHN (mean: 237.073 ± 5.25 VHN) with no significant differences among the groups (p = 0.16). The demineralizing solution significantly decreased EMH in all of the groups (all, p < 0.001). EMH values was not significantly different among groups after demineralization (p = 0.869). All intervention groups (Group 1–4) showed a significant increase in %REMH values compared to demineralization (all, p < 0.001). Group 3 had the highest recovery in EMH values of all the intervention groups (p < 0.05). pH-cycling caused a decrease in %REMH for all of the groups compared to after intervention. Groups 2 and 5 had the lowest %REMH reduction after pH-cycling; however, this difference between groups 2 and 5 was not significant (p = 0.993).

**Table 2:** Comparison of enamel surface microhardness in experimental groups.

Group	Condition (mean ± SD)					
	Baseline	DEM	Intervention	pH-cycling	%REMH intervention	%REMH pH-cycling
1	236.51 ± 6.52^A,a^	132.78 ± 5.93^A,b^	177.74 ± 5.67^c^	117.69 ± 5.91^d^	43.27 ± 3.57^A^	− 15.04 ± 9.01^A^
2	235.64 ± 3.2^A,a^	131.15 ± 4.49^A,b^	178.55 ± 6.64^c^	133.03 ± 6.26^d^	45.34 ± 4.79^A^	1.76 ± 6.22^B^
3	234.48 ± 4.77^A,a^	130.78 ± 4.20^A,b^	185.65 ± 5.23^c^	118.85 ± 5.70^d^	52.86 ± 4.79^B^	− 11.91 ± 9.11^A^
4	234.27 ± 5.57^A,a^	130.35 ± 5.77^A,b^	176.89 ± 6.72^c^	117.71 ± 9.46^d^	44.81 ± 4.25^A^	− 12.38 ± 11.23^A^
5	233.31 ± 5.47^A,a^	130.25 ± 6.19^A,b^	177.23 ± 4.56^c^	129.82 ± 7.59^d^	45.62 ± 3.85^A^	− 0.49 ± 7.39^B^
6	230.12 ± 6.83^A,a^	130.12 ± 4.14^A,b^	130.09 ± 3.84^c^	76.08 ± 6.18^d^	− 0.1 ± 5.4^C^	− 54.17 ± 6.23^C^
p-value	0.1689	0.8690	< 0.001	< 0.001	< 0.001	< 0.001

*%REMH* Percentage of enamel microhardness (EMH) recovery, *%REMH pH-cycling* Percentage of EMH recovery after pH-cycling (n = 12), *SD* Standard deviation.

In each row, means with the same lowercase letter are not significantly different (within-group analysis).

In each column, means with the same capital letter are not significantly different (between-group analysis).

Statistical significance: p < 0.05.

Group 1: Phosphoric acid + Clinpro™; group 2: Phosphoric acid + Aegis®; group 3: Icon®; group 4: Hydrochloric acid + Clinpro™; group 5: Hydrochloric acid + Aegis®; group 6: Control.

**Figure 3:**
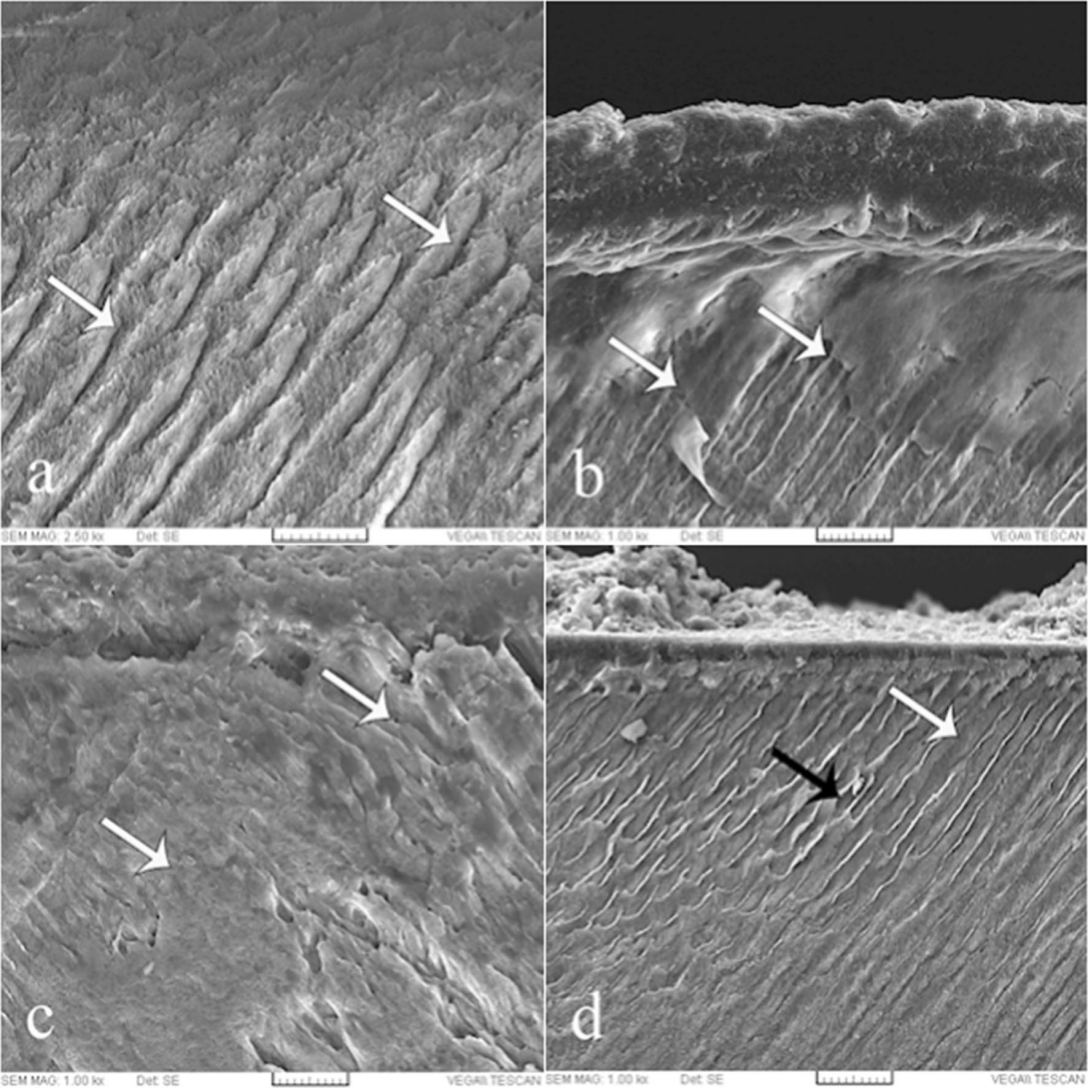
Scanning electron microscope image of the enamel after: (**a**) Demineralization (control group) (arrows), (**b**) Fissure sealant penetration and interface with the enamel treated with 37% phosphoric acid (group 1) (arrows), (**c**) Infiltrant resin penetrated into deeper areas and disperses across a larger area (arrows) (group 3), (**d**) Fissure sealant penetration and interface with the enamel treated with 15% hydrochloric acid (group 4). White arrow shows infiltrated area and black arrow shows non-infiltrated area. Magnification: 2500 × and 1000 ×.

The SEM findings revealed that demineralization resulted in dissolution of the enamel core and peripheral enamel rod, all of which led to the generation of spaces in the enamel. IR penetrated into deeper areas compared to the sealants. Penetration of FS on the surface treated with hydrochloric acid was greater than in the phosphoric acid groups. However, there were some unfilled spaces observed in groups 4 and 5 (Fig. 3).

## Discussion

The aim of the current study was to compare the effectiveness of two microinvasive methods to infiltrate and change the EMH of non-cavitated early caries lesions. We observed that all materials used on the treated enamel surfaces penetrated the demineralized enamel and increased %REMH, which supported the findings of previous studies^4, 8, 16^.

High resolution images and CLSM contrast allow researchers to analyze the ability of various materials to penetrate biological samples^17^. We used this accurate technique based on the findings from earlier reports^4, 7, 18, 19^. Image analysis obtained with CLSM demonstrated higher %PP after IR and fluoride FS along with hydrochloric acid pretreatment. Our result showed that IR had higher penetration than the FS associated with phosphoric acid surface preparation, which agreed with previously reported results^4, 8, 20, 21^. This might be related to the characteristics of the materials such as penetration coefficient and the surface pretreatment methods^4, 7^.

The penetration coefficient includes viscosity of the material, enamel surface tension, and “contact angle” between the liquid and tooth surface^22^. Enamel pretreatment with hydrochloric acid might lead to greater penetration by IR and sealant. During the early stages of caries formation, the remineralization process leads to a thick highly mineralized enamel surface layer, which creates a resistant barrier to material penetration. Hydrochloric acid (15%) more efficiently erodes the superficial enamel surface compared to 37% phosphoric acid^4, 6^. This might explain the deeper penetration by IR or fluoride FS along with hydrochloric acid, given that both materials mainly consist of resin. In addition, phosphoric acid more readily reduces enamel permeability than hydrochloric acid^23^. Other factors that influence penetration include surface dryness and the application time^24, 25^. The presence of water within the body of the lesion might interact with resin penetration. For this purpose, we applied ethanol before IR to remove excess moisture. In addition, IR consists of triethylene glycol dimethacrylate which increases resin penetration^4^.

In the present study, the ACP sealant had more superficial infiltration compared to IR, even after the surface was etched with hydrochloric acid. This might be related to the higher molecular weight of the resin (urethane dimethacrylate) and high filler content of the ACP sealant compared to the IR and fluoride sealants. Clinpro™ is a resin-based fluoride FS that consists of bisphenol A glycidyl methacrylate and it has a low viscosity, high flow, and wettability and is filler-free^26, 27^. The sealant did not show any significant difference with IR after enamel pretreatment with hydrochloric acid.

SEM images revealed the presence of enamel demineralization and etching, which led to dissolution of the organic materials, increased surface roughness, and the creation of voids and spaces due to loss of prism and interprism of the enamel. IR penetrated into deeper areas compared to the sealants, especially in samples where their surfaces were treated with phosphoric acid. IR disperses across a larger area and it produces a homogenous layer. The results confirmed our CLSM images. Some researchers reported the penetration of IR in demineralized early caries lesion, which supported the current study results^3, 28^.

The microhardness test is a simple laboratory test used to assess tooth microstructure changes after interventions. We followed the cross-sectional EMH test protocols, which were used by previous studies^18, 29^ instead of surface EMH and the designed method was based on a pilot study. FS cannot deeply penetrate enamel, as seen with IR. This hard layer of FS over the enamel did not permit the indenter of the microhardness device to reach the demineralized enamel located beneath the FS. Like others, we also observed that the interventions had higher %REMH compared to the control group, which indicated the effectiveness of the microinvasive methods to improve EMH. This finding supported the results of other studies^7, 8, 16, 30^. Our results showed a higher %REMH after IR compared to the sealants, which was in accordance with previous study^8^. These results may be related to the features of IR.

In the present study, we simulated in vivo conditions by performing pH-cycling. Overall, there was a reduction in %REMH after pH-cycling. The change in %REMH value after pH-cycling in the ACP sealant was lower than the other groups. This might be related to the composition of FS because it contains ACP, as smart materials that release ions under acidic conditions^30^. Clinpro™ FS releases a limited amount of fluoride ions and these ions cannot penetrate deeply. ACP has a higher mineral content than the fluoride sealant and leads to hydroxyapatite formation^16,31–34^. These factors might decrease %REMH in the ACP sealant groups after pH-cycling. IR did not contain remineralizing agents; after carrying out the acidic challenge, it absorbed water and was deleterious^3^. These factors might reduce the %REMH of IR, which supported the findings of one study^35^.

Our study had some limitations. The in vitro analysis did not provide the same conditions as a clinical setting; our sample size was limited; and there was no similar study on primary teeth to compare our results. Therefore, we compared our results with reports on permanent teeth. Primary teeth have higher porosity than permanent teeth, and this may lead to faster progression of caries lesions in these teeth. We also followed the manufacturer's instructions and used accurate methods of assessment with one operator. The results of the present study showed the effectiveness of IR and fluoride resin-based sealant in early caries lesions. Therefore, we suggest that more in vitro and in vivo studies should be performed to assess the capability of these materials to prevent ECC in primary teeth.

## Conclusion

Microinvasive interventional methods that use sealants and infiltrated resin on early lesions led to penetration of the materials into the subsurface layer and varying degrees of increased EMH. The infiltrated resin, followed by the fluoride resin-based sealant along with surface treatment with hydrochloric acid had a higher percentage of penetration. Although this criterion in the ACP sealant was less than the other two materials, the ACP sealant had the lowest %REMH reduction after the pH-cycling process. Due to the limited availability and high price of IR, the use of FSs to treat non-cavitated primary teeth along with surface pretreatment with hydrochloric acid should be considered.

## Data Availability

The datasets generated during and/or analyzed during the current study are available from the corresponding author upon reasonable request.
